# 18722 Association between neighborhood overcrowdedness, multigenerational households, and COVID-19 in New York City

**DOI:** 10.1017/cts.2021.589

**Published:** 2021-03-30

**Authors:** Arnab K. Ghosh, Sara Venkatraman, Orysya Soroka, Evgeniya Reshetnyak, Mangala Rajan, Anjile An, John K. Chae, Christopher Gonzalez, Jonathan Prince, Charles DiMaggio, Said Ibrahim, Monika M. Safford, Nathaniel Hupert

**Affiliations:** 1 Department of Medicine, Weill Cornell Medical College, Cornell University, 525 E 68th St., New York, NY, USA 10065; 2 Department of Statistics and Data Science, Cornell University, 129 Garden Ave., Ithaca, NY, USA 14853; 3 Department of Population Health Sciences, Weill Cornell Medical College, Cornell University, 402 E 67th St., New York, NY USA 10065; 4 Silberman School of Social Work at Hunter College, City University of New York, 2180 Third Ave, New York, NY USA 10035; 5 Department of Surgery, New York University School of Medicine, 462 First Ace, NBV 15, New York, NY USA 10016

## Abstract

ABSTRACT IMPACT: Patients living in overcrowded zip codes were at increased risk of contracting severe COVID-19 after controlling for confounding disease and socioeconomic factors OBJECTIVES/GOALS: This study sought to examine whether residences in over-crowded zip codes with higher reported over-crowding represented an independent risk factor for severe COVID-19 infection, defined by presentation to an emergency department. METHODS/STUDY POPULATION: In this zip code tabulated area (ZCTA)-level analysis, we used NYC Department of Health disease surveillance data in March 2020 merged with data from the CDC and ACS to model suspected COVID-19 case rates by zip code over-crowdedness (households with greater than 1 occupant per room, in quartiles). We defined suspected COVID-19 cases as emergency department reported cases of pneumonia and influenza-like illness. Our final model employed a multivariate Poisson regression models with controls for known COVID-19 clinical (prevalence of obesity, coronary artery disease, and smoking) and related socioeconomic risk factors (percentage below federal poverty line, median income by zip-code, percentage White, and proportion of multigenerational households) after accounting for multicollinearity. RESULTS/ANTICIPATED RESULTS: Our analysis examined 39,923 suspected COVID-19 cases across 173 ZCTAs in NYC between March 1 and March 30 2020. We found that, after adjusted analysis, for every quartile increase in defined over-crowdedness, case rates increased by 32.8% (95% CI: 22.7%% to 34.0%, P < 0.001). DISCUSSION/SIGNIFICANCE OF FINDINGS: Over-crowdedness by zip code may be an independent risk factor for severe COVID-19. Social distancing measures such as school closures that increase house-bound populations may inadvertently worsen the risk of COVID-19 contraction in this setting.

